# Correlation of Vitamin D Levels with Pigmentation in Vitiligo Patients Treated with NBUVB Therapy

**DOI:** 10.1155/2014/493213

**Published:** 2014-03-23

**Authors:** Manu Sehrawat, Tarlok Chand Arora, Amrita Chauhan, Hemanta Kumar Kar, Amitabh Poonia, Vijayeeta Jairath

**Affiliations:** ^1^PGIMER, Dr. RML Hospital, New Delhi, India; ^2^Pt. B.D. Sharma Post Graduate Institute of Medical Sciences, Rohtak, Haryana 124001, India

## Abstract

Cholecalciferol (vitamin D) might play a physiological role in photo-induced melanogenesis in human skin. We estimated the levels of 25-hydroxy vitamin D [25(OH)D] before, during, and after Narrow Band Ultraviolet B (NBUVB) radiation in patients of vitiligo and their correlation with NBUVB induced pigmentation. Thirty patients of vitiligo and equal number of age and sex matched controls were recruited for the study. Vitiligo patients were treated with NBUVB thrice weekly for 12 weeks. [25(OH)D] levels and Vitiligo Area and Severity Index (VASI) were calculated at 0 (baseline), 6, and 12 weeks. Baseline [25(OH)D] levels were measured in controls. Significant reduction in VASI score was observed after 12 weeks of therapy. Comparison and correlation between mean improvement in VASI and [25(OH)D] levels at 12 weeks showed moderate correlation, and the results were statistically insignificant. Mean reduction in VASI and increase in [25(OH)D] levels after 12 weeks of NBUVB showed moderate correlation. Thus, vitamin D might play a significant role in photo-induced melanogenesis. However, there might be additional effects of the phototherapy on melanogenesis. The complete mechanism of NBUVB induced pigmentation in vitiligo needs to be elucidated.

## 1. Introduction

Vitiligo is characterized by complete but selective loss of melanocytes from the interfollicular epidermis [1].

Convergence theory proposes that autoimmune diathesis, genetic factors, defective free radical defence, accumulation of neurochemical substances, chemicals (4-tertiary butyl phenol), psychological factors like stress, and an intrinsic defect of the function of melanocytes probably contribute in variable proportions to the destruction of pigment cells [2]. Recently, there has been growing interest in the role of vitamin D_3_ in the pathomechanism of vitiligo and its relevance in the treatment of vitiligo.

A significant body of data suggests that vitamin D_3_ is strongly immunosuppressive and that low levels are associated with autoimmune conditions including vitiligo. However, the cause of low vitamin D_3_ in patients with autoimmune conditions remains unknown [3]. Further, Tomita et al. showed that vitamin D_3_ increased the tyrosinase content of cultured human melanocytes [4]. These findings suggest a possible role of vitamin D_3_ in modulating melanogenesis.

Narrow Band Ultraviolet B (NBUVB) therapy has found favor amongst dermatologists since its first use by Westerhof and Nieuweboer-Krobotova in the treatment of vitiligo in 1997 because of its safety and efficacy. Nevertheless, the mechanism of NBUVB induced pigmentation has remained an enigma. Well-documented immunomodulatory effects of UV radiation explain the stabilization of the local and systemic abnormal immune responses [5]. NBUVB increases synthesis of IL-1 (interleukin-1), TNF-*α* (Tumour necrosis factor-*α*), and LTC-4 (Leucotriene C-4) and these cytokines induce melanocyte mitogenesis, melanogenesis, and melanocyte migration. Moreover, the current data demonstrates that the UVB portion of the sunlight (290–320 nm) brings about the photochemical conversion of 7-dehydrocholesterol to previtamin D_3_ in the stratum spinosum and stratum basale, which is the key step to vitamin D_3_ synthesis. Since vitamin D_3_ may act to induce immature melanocytes in the bulge region of hair follicles to produce melanin by stimulating their differentiation and their expression of endothelin B receptor [6], we posit that at least a part of NBUVB induced repigmentation in vitiligo may be explained by NBUVB induced vitamin D_3_ synthesis. Therefore, we sought to evaluate the vitamin D status in vitiligo patients before, during, and after NBUVB therapy and correlated it with repigmentation.

## 2. Materials and Methods

The study included 30 patients of generalized vitiligo (≥5% body surface area involved) of 18–45 years of age from the outpatient department of our institute with due consent. Patients who had taken treatment for vitiligo in the last 8 weeks, had coexistent diabetes mellitus, thyroid disorder, skin, or any other malignancy or photosensitivity, had history of previous intolerance or failure of phototherapy, or were taking any known photosensitizer drug were excluded from the study. An equal number of age and sex matched consenting healthy volunteers were recruited as controls for baseline vitamin D levels.

Baseline (week 0) serum 25-hydroxy vitamin D levels were measured in both study group and control group. Subsequently, 25-hydroxy vitamin D levels were measured at 6 weeks and 12 weeks in the study group (i.e., vitiligo patients on NBUVB therapy). Two mL of fasting morning venous blood sample was drawn for 25-hydroxy vitamin D level estimation which was done by Competitive Enzyme Linked Immunosorbent Assay (ELISA) technique using Immunodiagnostic systems (ids) 25-hydroxy vitamin D enzyme immunoassay (EIA) kit.

NBUVB phototherapy was given using Waldmann UV 1000 L (TL 01) machine. The irradiation dose was started at a dose of 0.3 J/cm^2^ (minimum erythema dose of Indian skin) [7] and the dose was increased by 20% on each subsequent visit till just faint erythema appeared. If symptomatic erythema (burning, pain) or blisters developed, phototherapy was withheld till the lesions healed. Then irradiation was again started at a dose 20% lesser than the dose which induced erythema or blisters. Thereafter the dose was increased by 10% on subsequent visits [8]. Phototherapy was given thrice weekly on nonconsecutive days for 12 weeks. During each treatment genitals were shielded and eyes were protected with UV safety glasses. All patients were examined at 6 weeks interval, that is, week 0 (baseline), week 6, and week 12 (end of therapy) and Vitiligo Area Severity Index (VASI) was calculated on each of these three occasions (vide infra) by the same physician.

The degree of depigmentation was measured by VASI determined by the product of the area of vitiligo in hand units (set as 1% per unit) and the extent of depigmentation within each hand unit-measured patch [9].

Each patch was taken separately and then the extent of residual depigmentation within each affected patch was estimated to the nearest of one of the percentages (Table 1):

(1) VASI  =∑All  body  sites(Hand  Units)       ×(Residual  Depigmentation).

VASI may have a range between 0–100.

Improvement in VASI score was determined by the treatment evaluation criterion [10] as shown in Table 2.

Colour of pigmentation was compared with the colour of the patient's normal skin and was classified as [11]:

1. somewhat darker,
2. somewhat lighter,
3. same.

Type of repigmentation was classified as [12]:

1. predominantly perifollicular,
2. predominantly marginal,
3. predominantly diffuse,
4. combined.

Side effects like erythema, pruritus, blisters, and so forth were recorded at each visit.

Statistical analysis was done using paired *t*-test, unpaired *t*-test, and Spearman's correlation (*ρ*). *P* value of 0.05 or less was considered as significant in all statistical tests. Correlation (*ρ*) value of less than 0.3 was considered as weak, value ranging from 0.3 to 0.7 was taken as moderate, and value more than 0.7 was taken as strong correlation.

## 3. Results

The study comprised 20 females and 10 males. Male, female ratio was 1 : 2. Five patients had phototype IV and 25 patients had phototype V skin. Mean age of the patients was 31.33 ± 7.73 years. The youngest patient was 19 years and the eldest was 42 years at the time of inclusion in the study. Maximum number of patients had onset of disease between 11–20 years of age and approximately 76.66% (*n* = 23) patients had onset before 30 years of age. The mean age of onset was 20.33 years. Duration of disease ranged between 8 months and 35 years with a mean of 10.47 years. Maximum number of patients presented within 10 years (*n* = 20) of onset and majority of them were females (*n* = 13). Twenty-five percent (*n* = 6) of the patients with vitiligo had positive family history.

24 (80%) patients had deficient (<25 nmol/L) and 6 (20%) had insufficient baseline levels (25–75 nmol/L) of 25(OH)D. None of the patients had normal (75–250 nmol/L) levels. Levels of 25(OH)D in the control group were also found to be low. Ninety percent (*n* = 27) of the controls had insufficient (25–75 nmol/L) levels and 10% (*n* = 3) had deficient (<25 nmol/L) 25(OH)D levels. None of the subjects in the control group too had normal (75–250 nmol/L) levels. Mean baseline level of 25(OH)D in patients was 19.80 ± 10.47 nmol/L, while that of controls was 31.51 ± 5.62 nmol/L. Although levels of 25(OH)D were low in patients of vitiligo as well as in controls, the levels were significantly lower in the vitiligo patients (*P* ≤ 0.001). (Tables 3 and 4).

Baseline mean Vitiligo Area Severity Index (VASI) score was 21.66 ± 17.21. Correlation between mean VASI and mean 25(OH)D levels was weak (correlation coefficient (*ρ*) = 0.091) among vitiligo patients and was statistically insignificant (*P* = 0.631) before starting the phototherapy. After 6 weeks and 12 weeks of phototherapy, VASI scores showed reduction, that is, increase in repigmentation (Tables 5 and 6). Levels of 25(OH)D at 6 and 12 weeks showed significant improvement (Table 6). Comparison and correlation between mean improvement in VASI and 25(OH)D at 6 and 12 weeks showed weak and moderate correlation, respectively, but the results were statistically insignificant (Tables 7 and 8).

Average cumulative dose of NBUVB phototherapy after 6 weeks was 13.45 ± 7.1 J/cm^2^ given in 14.53 ± 1.9 number of treatment sessions and the average pigmentation achieved was 10–25%. After 12 weeks, 36.71 ± 19.87 J/cm^2^ of NBUVB phototherapy was given in 30.87 ± 2.61 treatment sessions and showed 25–50% repigmentation (much improved grade).

**Figure 1:**
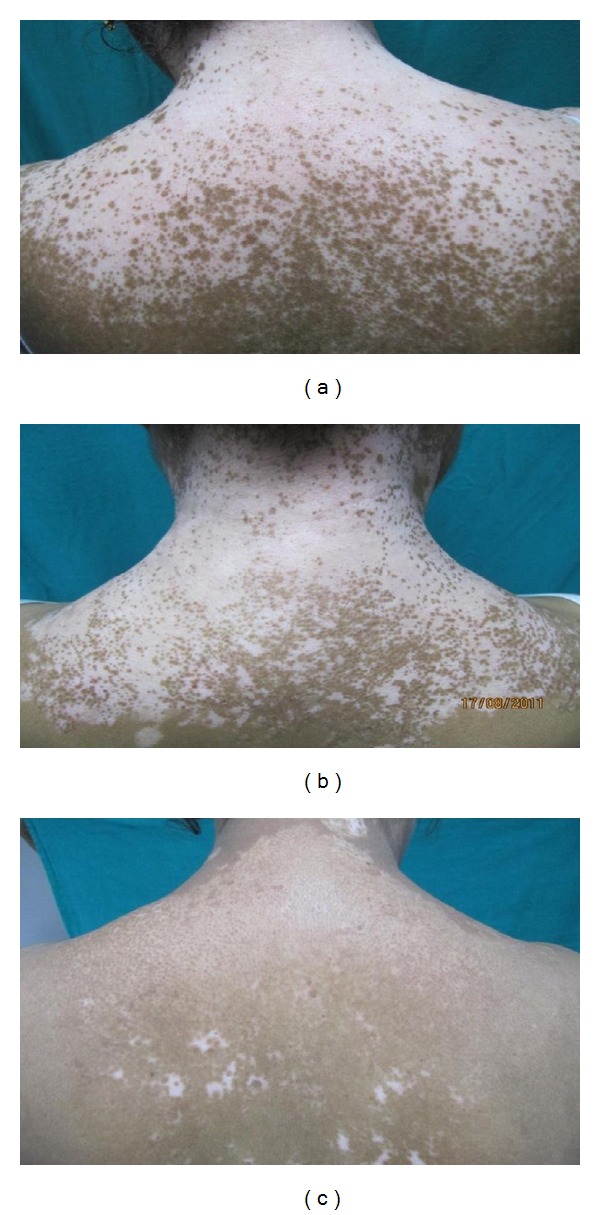
Patient of vitiligo treated with NBUVB phototherapy at baseline (0 week), after 6 weeks, and 12 weeks of phototherapy. The pigmentation appearing is perifollicular and that too of similar colour as that of the surrounding skin.

**Figure 2:**
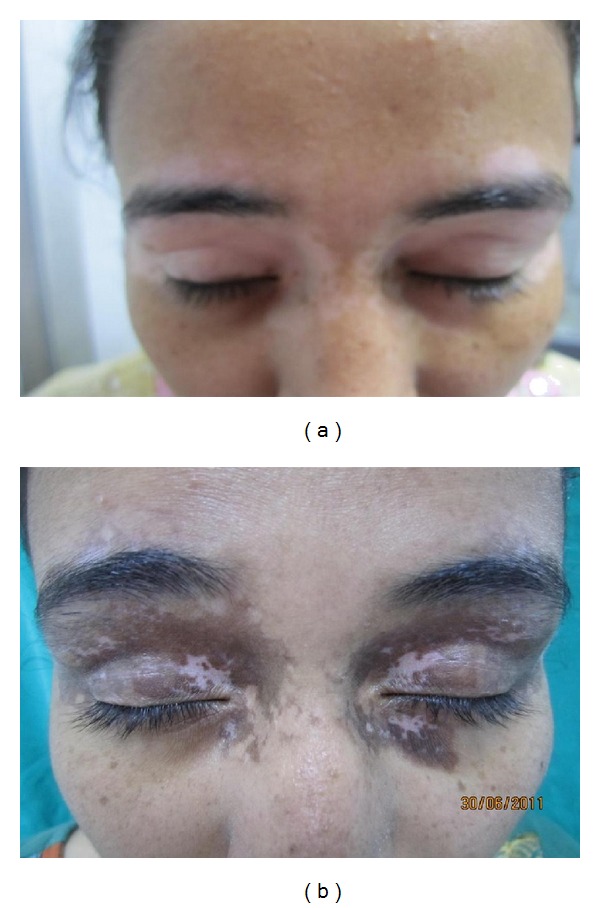
Patient of vitiligo treated with NBUVB phototherapy, showing diffuse type and somewhat darker colour of repigmentation after 12 weeks of therapy.

There was a good colour match of the treated area of vitiligo with the surrounding normal skin with NBUVB phototherapy as 22 (73.33%) patients showed same colour of initial repigmentation in vitiligo patches as that of the surrounding skin. The pattern of repigmentation started as predominantly perifollicular and was observed in 25 (83.33%) patients. The other patterns of repigmentation noticed were predominantly diffuse, predominantly marginal, and combined type of repigmentation, that is, perifollicular, as well as marginal. (Figures 1 and 2) Seventy percent (*n* = 21) of the patients had no side effects with phototherapy. Most common side effect observed was marked erythema in 5 (16.66%) patients, blistering in 3 (10%), pruritus in 4 (13.33%), and only 1 patient developed acneiform eruption.

## 4. Discussion

Approximately, 0.1–2% of the world's population is currently affected by vitiligo [13]. Various factors have been implicated in the etiopathogenesis of vitiligo including the role of calcium imbalance [14], vitamin-D receptor-Apa-1 polymorphism [15], and low levels of circulating 25-OH vitamin D [16]. Interestingly, patients receiving NBUVB have shown an increase in the levels of 25(OH) vitamin D [17]. Moreover, molecular studies have shown that vitamin D increases the tyrosinase content of melanocytes [4] and induces immature melanocytes in the bulge region of hair follicles to produce melanin [6]. Hence, vitamin D at cellular level modulates melanogenesis. Whether the circulating levels of 25(OH) vitamin D show any change with successive NBUVB sessions and whether these levels correlate or translate into clinical repigmentation is the point of interest of the current paper.

The present study included Indian subjects with skin phototype IV and V. None of the vitiligo patients and control subjects had normal baseline levels of vitamin D. The finding reiterates the observations made by other authors from the Indian subcontinent wherein a large population of healthy people (60%–70%) was noted to have low levels of circulating 25(OH) vitamin D [18, 19]. The observations probably support the Loomis theory that melanin pigmentation acts as a major determinant in limiting cutaneous vitamin D formation [20]. Moreover, fortification of food with vitamin D_3_ not being a norm in India may be an important contributing factor to low serum levels of 25(OH) vitamin D levels in Indian population. However, 25(OH) vitamin D levels were lower in vitiligo patients as compared to the control subjects.

**Figure 3:**
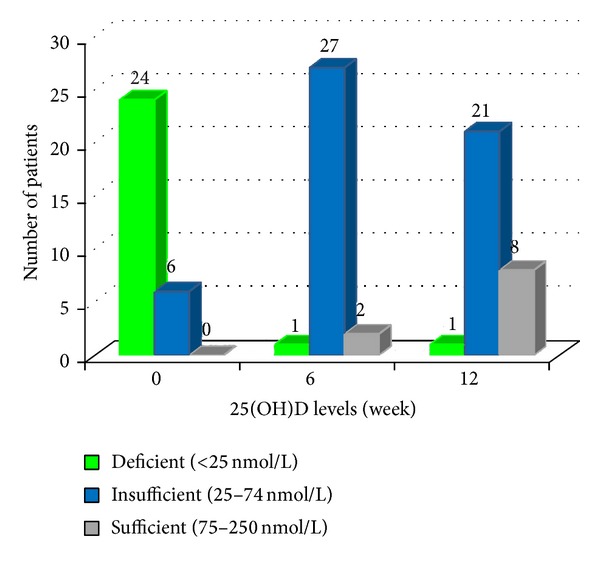
Levels of 25(OH)D at 0, 6, and 12 weeks of phototherapy in vitiligo patients.

The results of our study show that levels of 25(OH) vitamin D increased significantly with increase in the cumulative dose of NBUVB. Also, VASI scores showed improvement with increase in the cumulative dose of NBUVB. Comparison and correlation between mean improvement in VASI and 25(OH) vitamin D at 6 weeks showed weak correlation. However, the value of correlation coefficient increased with increase in the cumulative dose of NBUVB and the correlation became moderate at 12 weeks. Based on these results, further increase in correlation may be speculated. The current findings, however, do not adequately support the significant role of vitamin D in photo-induced melanogenesis. Although it can only be conjectured that the repigmentation in vitiligo by NBUVB may partly be mediated by vitamin D_3_ induced stimulation of melanogenesis in view of the moderate correlation noted at 12 weeks of phototherapy. To further support the point, 2 cases merit special mention. One patient showed increase in VASI during the first 6 weeks and then decrease in VASI in the next 6 weeks. Her 25(OH)D levels did not change significantly during this period. At 0 week, 6 weeks, and 12 weeks her vitamin D levels were 13.22, 14.14, and 16.05 nmol/L, respectively, and the corresponding VASI was 24.95%, 25.42%, and 23.10%. Another patient showed improvement during the first 6 weeks but worsened in the next 6 weeks. However, worsening did not reach pretreatment levels. Her VASI at 0, 6, and 12 weeks was 47.8%, 39.84%, and 40.87%, respectively. Along with improvement in VASI there was an increase in 25(OH)D levels from 11.13 to 80.73 nmol/L from 0 to 6 weeks (Figure 3). In the next 6 weeks, the pigmentation decreased. VASI increased from 39.84% to 40.87%. Along with worsening of VASI there was corresponding decrease in the 25(OH)D levels in that patient from 80.73 nmol/L to 41.20 nmol/L. Depigmentation coincided with decrease in 25(OH) vitamin D and an increase in vitamin D levels correlated clinically with repigmentation in these patients. Important point to note here is that nonresponders failed to synthesize adequate vitamin D inspite of being on NBUVB. Important point to note here is that nonresponders failed to synthesize adequate vitamin D. Thus, vitamin D seems to be a key position holder in NBUVB induced melanogenesis. Nevertheless, a large sample size is required to prove conclusively the role of vitamin D wherein such nonresponders can serve as controls and some statistical correlation can be arrived at.

Though a few studies show that NBUVB increases circulating levels of 25-hydroxy vitamin D [21, 22], there is yet no published study comparing the levels of 25(OH)D in patients of vitiligo before and after NBUVB phototherapy and correlating it with repigmentation.

Repigmentation achieved matched with the normal skin in 22 (73.33%) patients. 5 patients (16.66%) showed somewhat darker and 3 (10%) patients showed somewhat lighter colour in the patches of vitiligo after NBUVB phototherapy. This is in concordance with the results of earlier studies [11, 23, 24].

Most of the patients had a perifollicular pattern of repigmentation indicating that melanin is produced by the melanocytes in hair follicles. Vitamin D may act to induce immature melanocytes in hair follicles to produce melanin by stimulating their differentiation and their expression of EDNRB [6]. The downside of the study is its small sample size and short duration of study.

To conclude, the present study shows that there is no statistically significant correlation between reduction of VASI and 25(OH) vitamin D levels. However, the correlation is getting stronger with duration of phototherapy. We can only conjecture that vitamin D might play a role in view of the moderate correlation noted at 12 weeks of NBUVB therapy. Further studies are needed in this respect.

## What Is Known?

1. NBUVB therapy is a safe and efficacious therapy for the treatment of vitiligo.
2. NBUVB therapy increases 25-hydroxy vitamin D levels.

## What Is New?

Vitamin D might have a significant role in NBUVB induced repigmentation of vitiligo as the correlation between repigmentation of vitiligo lesions and vitamin D levels increased with increase in duration of phototherapy.

## Figures and Tables

**Table 1:** Evaluation of depigmentation in a vitiligo patch.

% Depigmentation	Clinical evaluation of pigmentation
100% depigmentation	No pigment is present
90% depigmentation	Specks of pigment are present
75% depigmentation	Depigmented area exceeds the pigmented area
50% depigmentation	Depigmented and pigmented areas are equal
25% depigmentation	Pigmentad area exceeds the depigmented area
10% depigmentation	Only specks of depigmentation are present

**Table 2:** Treatment evaluation criterion based upon the VASI score.

VASI score	>−50	Very much worse
VASI score	−50 to −25	Much worse
VASI score	−25 to −10	Worse
	Change in VASI score	Grade
VASI score	−10 to 0	Minimally worse
VASI score	0 to +10	Minimally improved
VASI score	+10 to 25	Improved
VASI score	+25 to 50	Much improved
VASI score	>+50	Very much improved

**Table 3:** Baseline levels of 25(OH)D in patients of vitiligo and controls.

25(OH)D	Number of patients	Controls
Deficient (<25 nmol/L)	24	3
Insufficient (25–74 nmol/L)	6	27
Normal (75–250 nmol/L)	0	0

Total	30	30

**Table 4:** Comparison of mean of baseline 25(OH)D levels in patients of vitiligo and controls.

25(OH)D levels	Number of patients	Mean ± SD	*P* value
		(ng/mL)	
Patients	30	19.80 ± 10.47	<0.001
Controls	30	31.51 ± 5.62	

**Table 5:** Comparison and correlation between mean VASI and mean 25(OH)D before starting phototherapy.

Before therapy	Number of patients	Mean	Spearman's correlation (*ρ*)	*P* value
VASI	30	21.66 ± 17.21	0.091	0.631
25(OH)D	30	19.80 ± 10.47 nmol/L		

**Table 6:** Change in VASI after 6 and 12 weeks of NBUVB phototherapy.

Change in VASI (%)	Number of patients at 6 weeks	Number of patients at 12 weeks
Very much worse (>−50)	0	0
Much worse (−50 to −25)	0	0
Worse (−25 to −10)	0	0
Minimally worse (−10 to 0)	2	0
Minimally improved (0 to +10)	3	3
Improved (+10 to +25)	14	5
Much improved (+25 to +50)	10	14
Very much improved (>+50)	1	8

Total	30	30

**Table 7:** Comparison and correlation between mean improvement in VASI and 25(OH)D after 6 weeks of phototherapy.

After 6 weeks	Number of patients	Mean improvement (%)	Spearman's correlation (*ρ*)	*P* value
VASI	30	20.95 ± 13.12	0.287	0.124
25(OH)D	30	187.09 ± 158.44		

**Table 8:** Comparison and correlation between mean improvement in VASI and 25(OH)D after 12 weeks of phototherapy.

After 12 weeks	Number of patients	Mean improvement (%)	Spearman's correlation (*ρ*)	*P* value
VASI	30	36.66 ± 17.22	0.301	0.107
25(OH)D	30	260.59 ± 181.45		
